# Supplementary material for: Effects of childhood adversity on socially learned placebo analgesia in virtual reality: A cross-sectional study
Source: medRxiv. 2025 Dec 9:2025.12.04.25341572. Preprint. [Version 1] doi: 10.64898/2025.12.04.25341572 (PMC12704645; doi:10.64898/2025.12.04.25341572)
Supplement: Supplement 1 [file media-1.docx]

**Flow chart**:


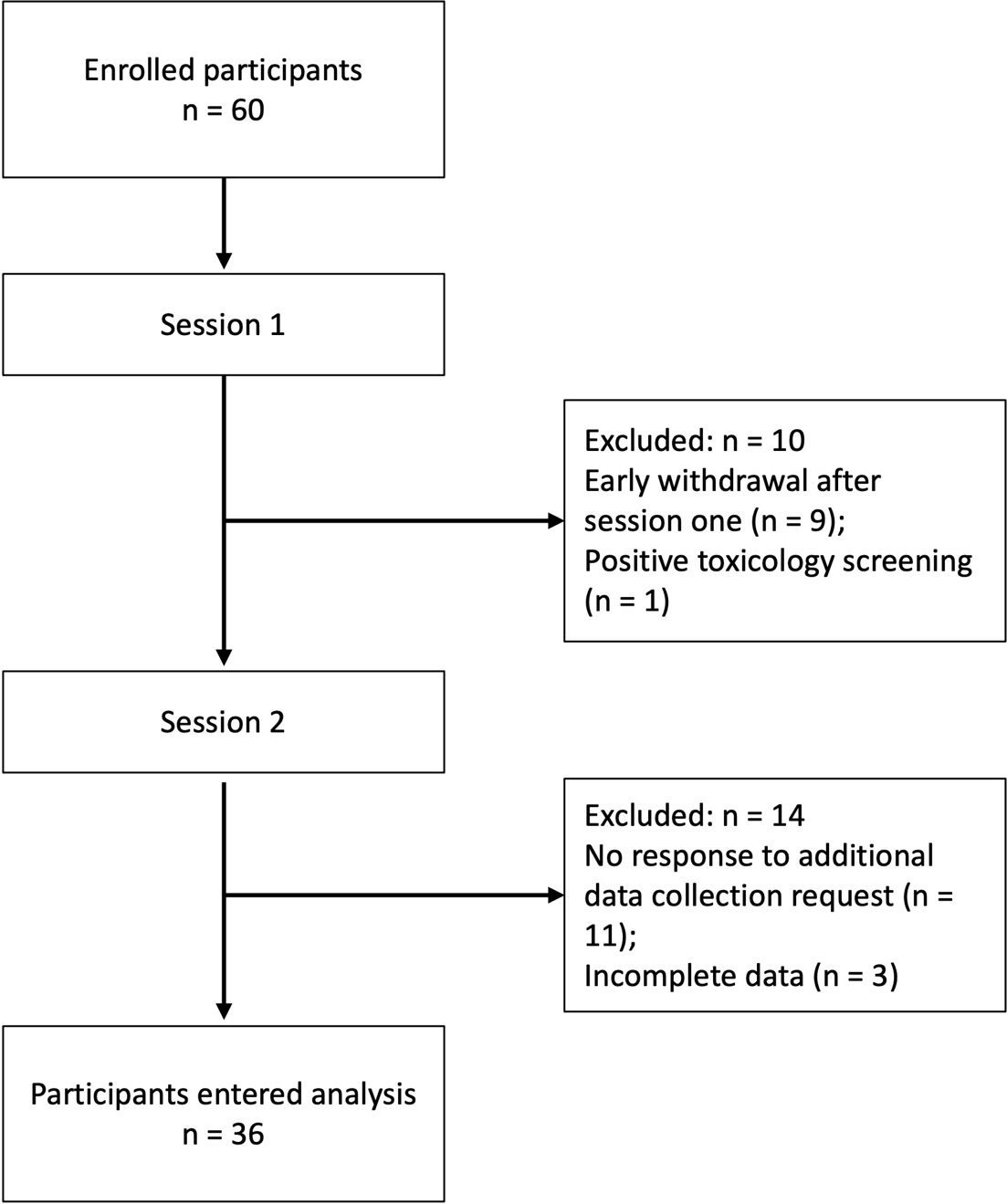


Twenty-four participants were excluded from protocol for early withdrawal after session one (n = 9); a positive toxicology screening (n = 1); incomplete data (n = 3); and lack or response to request for additional data collection (n = 11).
